# Phosphorylation of Mutationally Introduced Tyrosine in the Activation Loop of HER2 Confers Gain-of-Function Activity

**DOI:** 10.1371/journal.pone.0123623

**Published:** 2015-04-08

**Authors:** Zexi Hu, Xiaobo Wan, Rui Hao, Heng Zhang, Li Li, Lin Li, Qiang Xie, Peng Wang, Yibo Gao, She Chen, Min Wei, Zhidong Luan, Aiqun Zhang, Niu Huang, Liang Chen

**Affiliations:** 1 College of Life Sciences, Beijing Normal University, Beijing, 100875, China; 2 National Institute of Biological Sciences, Beijing. Beijing, 102206, China; 3 BeiGene (Beijing) Co., Ltd., Beijing, 102206, China; 4 The Central Hospital of Lishui City, Zhejiang, 323000 China; 5 Fuzhou Pulmonary Hospital of Fujian, Fujian, 350008, China; 6 Beijing Ditan Hospital, Beijing, 100015, China; 7 Department of Thoracic Surgery, Cancer Institute and Hospital Chinese Academy of Medical Sciences, Beijing, 100021, China; 8 Liaoning Medical University, Jinzhou, Liaoning, 121001, China; 9 The General Hospital of People’s Liberation Army (301 Hospital), Fuxing Road, Beijing, 100853, China; 10 Collaborative Innovation Center of Cancer Medicine, National Institute of Biological Sciences, Beijing, Beijing, 102206, China; Fondazione IRCCS Istituto Nazionale dei Tumori, ITALY

## Abstract

Amplification, overexpression, and somatic mutation of the HER2 gene have been reported to play a critical role in tumorigenesis of various cancers. The HER2 H878Y mutation was recently reported in 11% of hepatocellular carcinoma (HCC) patients. However, its functional impact on the HER2 protein and its role in tumorigenesis has not been determined. Here, we show that HER2 H878Y is a gain-of-function mutation. Y878 represents a phosphorylation site, and phospho-Y878 interacts with R898 residue to stabilize the active conformation of HER2, thereby enhancing its kinase activity. H878Y mutant is transforming and the transformed cells are sensitive to HER2 kinase inhibitors. Thus, our study reveals the following novel mechanism underlying the tumorigenic function of the HER2 H878Y mutation: the introduction of a tyrosine residue into the kinase activation loop via mutagenesis modulates the conformation of the kinase, thereby enhancing its activity.

## Introduction

ErbB2 belongs to the ErbB family of receptor tyrosine kinases, which consists of ErbB1, ErbB2, ErbB3 and ErbB4, also known as EGFR, HER2, HER3 and HER4, respectively in humans. Members of the ErbB family play critical roles in normal cellular function and organismal development, as evidenced by the embryonic lethality exhibited by ErbB2 knockout mice [1] and the strain-dependent severe embryonic defects or post-natal lethality caused by EGFR knockout [2].

Although HER2 has no known ligand, it is a preferred dimerization partner for other ErbB family members. The activation of the ErbB receptor results in the autophosphorylation of its C-terminal tyrosine residues, which recruits signaling partners, including members of the Ras-Raf-MEK-MAPK pathway, PLC-γ1, phosphatidylinositol-3 kinase (PI3K)-AKT-S6 kinase (S6K), SRC, stress-activated protein kinases (SAPKs), members of the PAK-JNKK-JNK pathway and the signal transducers and activators of transcription (STATs) (reviewed in [3]).

In the clinic, the ErbB family members are important proto-oncogenes, and their deregulation is often associated with several cancer types. For example, HER2 amplification is observed in 30% of breast cancer patients [4]. In addition to amplification, intragenic insertional mutations of HER2 are observed in 4% of lung cancers [5], and its kinase domain mutations are observed in 5% of gastric carcinomas, 2.9% of colorectal carcinomas and 4.3% of breast carcinomas [6]. Currently, HER2 is among the most intensely investigated kinase drug targets.

Many HER2-targeting reagents have been developed for cancer treatment. Trastuzumab [7], and more recently, pertuzumab [8], are antibodies that have been approved by the FDA for the treatment of HER2-overexpressing breast cancer. Both antibodies can bind to the extracellular domain of HER2 to prevent the activation of its intracellular kinase activity. In addition to antibodies, multiple small molecule inhibitors of HER2 are in various stages of clinical trials, and several have been approved by the FDA. For example, lapatinib targets the inactive conformation of the ERBB2 kinase, blocking its kinase activity [9]. Recently, irreversible inhibitors, such as BIBW2992 and HKI-272, have been developed for clinical usage [10]. However, their efficacy varies among patients, which is due, in part, to the fact that some mutations might confer *de novo* tumor cell resistance to cognate targeting drugs, as exemplified by the L755S HER2 mutation to lapatinib [11].

Recently, HER2 H878Y mutation was reported in 11% of hepatocellular carcinoma (HCC) patients [12]. However, the impact of this mutation on HER2 functioning has not been studied. Successful treatment of HCC is severely limited by paucity of clinically proven drug targets. It’s therefore important to carefully study functional impact of H878Y mutation on HER2 and explore the clinical relevance of this mutant protein. We here report that H878Y is a gain-of-function mutation. This specific mutation renders tyrosine phosphorylation at Y878 of HER2, an event that can only occur on the mutant form of HER2. Phospho-Y878 forms a salt bridge with the adjacent R898 residue, thus stabilizing the active conformation of HER2. To our knowledge, this is the first report describing the mutagenic introduction of a tyrosine into the activation loop of a kinase that is phosphorylated to stabilize the active conformation of the kinase. Consistently, we found that the HER2 H878Y mutation is transforming and transformed cells are sensitive to treatment with HKI-272.

## Materials and Methods

### Cell lines

NIH-3T3 cells were purchased from the ATCC and cultured in Dulbecco’s Modified Eagle’s Medium (DMEM) containing 10% FCS (Hyclone). Beas2b (ATCC) cells were cultured in DMEM medium supplemented with 10% FBS (Gibco). AML12 (ATCC) cells were cultured in a 1:1 mixture of Dulbecco's modified Eagle's medium and Ham's F12 medium with 10% FBS, 0.005 mg/ml insulin, 0.005 mg/ml transferrin, 5 ng/ml selenium, and 40 ng/ml dexamethasone. WEHI-3B cells were cultured in RPMI 1640 medium supplemented with 10% FBS (Gibco). Ba/F3 cells were cultured in RPMI 1640 medium supplemented with 10% FBS and 10% WEHI-3B conditioned medium (filtered supernatant). Ba/f3 and WEHI-3B cell lines are gifts from Prof. Pasi A. Jänne (Harvard University) [13]. All of the cell culture media were supplemented with 10 mM glutamine and 1% penicillin and streptomycin and incubated at 37°C in a 5% CO_2_ incubator.

### Cell viability

HER2-transformed Ba/F3 cells (2 x 10^3^) were incubated with the indicated inhibitors or DMSO (as a vehicle control) for 3 days. Viability was assayed using the CellTiter-Glo luminescent cell viability assay (Promega). The resulting luminescent signals were recorded using a multimode plate reader (PerkinElmer). All of the inhibitors were purchased from Selleck Chemicals. Cell culture grade dimethyl sulfoxide (DMSO) was purchased from Sigma.

### Soft agar and stable cell line generation

For mammalian HER2 expression, the DNA fragment encoding wild-type hHER2 was inserted into pCAG-IN (CAG promoter-intron-IRES-Neomycin [14]) multiple cloning sites. Site-directed mutagenesis were performed with on the pCAG-wtHER2-IN plasmid for deriving HER2 mutant isoforms (H878Y). The linearized HER2 vectors were transfected into NIH-3T3, Beas2b, AML12 and Ba/F3 cells, which were then selected with 500μg/ml, 1200μg/ml, 700μg/ml and 800μg/ml G418 respectively. The transfected cells (1.5 × 10^4^) were seeded in 0.4% low melting point agarose (invitrogen) with 10% FCS or FBS and DMEM. Bottom agar consisted of DMEM with 0.6% agarose, and 10% FCS or FBS. Colonies was counted 1 month after seeding in soft-agar. Assays were carried in 6-replicate for each isoform. When necessary, single colony was transferred to 24-well plate to establish a stable cell line. The transfected Ba/F3 cell were deprived of IL-3 after selected with G418 for 12 days. The transformed cells were then seeded at one cell/well in a 96-well plate to establish a stable clone.

### Western blotting

All cells were lysed in RIPA buffer (Beyotime) supplemented with protease and phosphatase inhibitors (Roche). Following lysis, the cells were sonicated. Western blotting was performed using standard methods. The blots were probed with the following antibodies: phospho-HER2-Tyr1221/1222 (Epitomics), β-actin (Sigma), phospho-HER2-Tyr877, phospho- HER2-Tyr1248(p-HER2), HER2, PLCγ1, phospho-PLCγ1-Tyr783, PARP, STAT5, phospho- STAT5-Tyr694, AKT, phospho-AKT-Ser473 (pAKT), ERK1/2, phospho-ERK1/2-Thr202/Tyr204, S6, phospho-S6-Ser235/236 (pS6) and phosphor-4E-BP1-Thr37/46 (Cell Signaling Technology).

## Results

### H878Y mutant HER2 elicited stronger signal to transform cells

**Fig 1:**
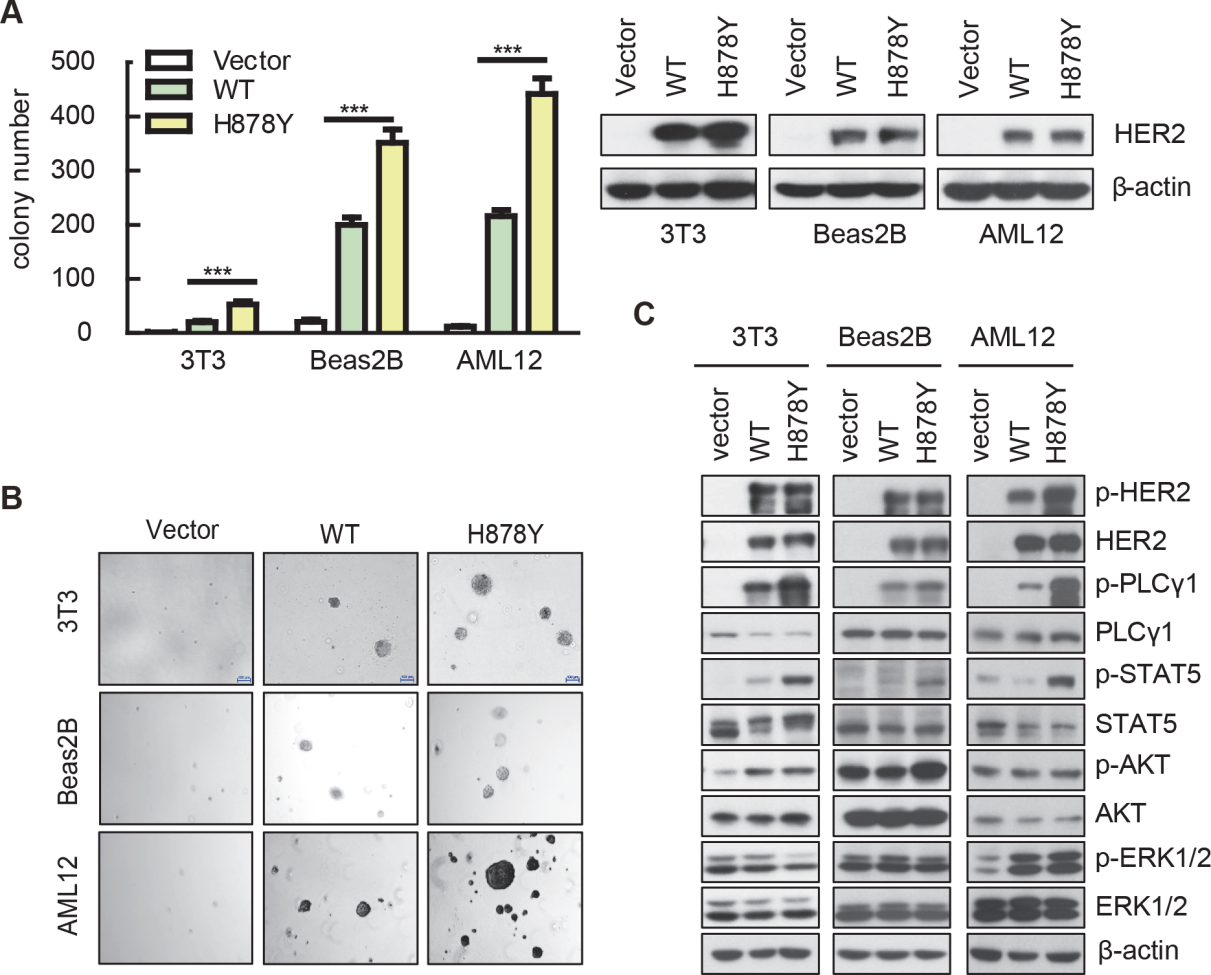
HER2 H878Yelicited stronger signal to transform cells. (A), (B) Soft-agar assay on cells transfected with control vector, WT, and H878Y HER2 mutant. (A) Left panel: Quantification and statistics of the soft-agar result. Colonies > 100 μm are counted. Values are mean ± SEM (n = 6). ***P<0.001. Right panel: Western blot was conducted to show the similar expression of HER2 proteins (B) Representative pictures of soft-agar colonies H878Y transformed cells form larger and more colonies on soft-agar assay. Pictures are photographed on day 30, Scale bars, 50μm. (C) Western blot analysis on NIH-3T3, Beas-2B and AML12 cells transfected with control vector, WT or H878Y constructs revealed H878Y could active canonical downstream signal.

H878Y mutant HER2 was identified in 11% of HCC patients and H878 locates in kinase domain [12], suggesting that it is a gain-of-function mutation. As treatment of HCC is limited by paucity of validated drug targets, it’s therefore highly interesting to study whether H878Y mutant HER2 plays an important role in HCC tumorigenesis. To test whether H878Y is a gain-of-function mutation, we overexpressed H878Y HER2 mutant (H878Y) in NIH-3T3 cells (referred to herein as 3T3 cells) and assayed its soft-agar colony forming capacity as readout of transforming ability side-by-side with wild type HER2 (WT HER2). These cells expressed similar level of HER2 proteins as revealed by Western analysis (right panel, Fig 1A). While control vector transfected cells formed no colonies, the WT isoform exhibited transforming ability in this soft-agar assay, consistent with previous reports [15]. In striking comparison, the H878Y mutant promoted a significant increase in the rate of soft-agar colony formation (p<0.01), and the colonies were larger (left panel, Fig 1A for bar graph and statistics, and 1B for representative pictures).

HER2 mutations were reported in a portion of lung cancer patients [16] and H878Y specifically in HCC [12]. We therefore checked transforming ability of H878Y in Beas-2B (a human bronchial epithelial cell line) and AML12 (a mouse hepatocyte cell line) respectively. Similarly, we found higher transforming ability of H878Y than WT in both cell lines (Fig 1A and 1B). These results indicate that H878Y is a gain-of-function mutation.

To investigate the HER2 protein elicited signaling underlying gain-of-function phenotype for H878Y mutant, we assessed the phosphorylation status of HER2, Erk and Akt, two typical downstream molecules [3]. Both the WT and H878Y HER2 isoforms were efficiently phosphorylated in 3T3, Beas-2B, and AML12 cells (Fig 1C). Interestingly, H878Y shows more robust phosphorylation in AML12 cells while similar level of phosphorylation was seen in 3T3 and Beas-2B cells. Phosphorylation of Akt in WT and H878Y was slightly higher than vector infected control in all 3 cell lines, with slightly stronger phospho-Akt in H878Y than WT cells. Phospho-Erk1/2 was similar between WT and H878Y in AML12 background. We also checked phosphorylation status for ErbB family signaling pathwaysand PLCγ1, an important downstream mediator of HER2 for cell growth and metastasis [17, 18]. Strikingly, PLCγ1 consistently showed higher phosphorylation in all 3 cell background. As signal transducer and activator (STAT) proteins has been reported to be important downstream mediators for HER2 [19, 20], we checked phosphorylation of STAT3 (data not shown) and STAT5 and found that STAT5 was much more strongly phosphorylated in cells transfected by H878Y than WT HER2 in all 3 cell background. WT HER2 transfected 3T3 and Beas-2B cells showed stronger STAT5 phosphorylation in comparison to vector transfected cells, while no significant difference was seen between vector and WT HER2 transfected AML2 cells (Fig 1C). These results therefore clearly showed that H878Y is a gain-of-function mutation.

### H878Y mutant HER2 has enhanced kinase activity

**Fig 2:**
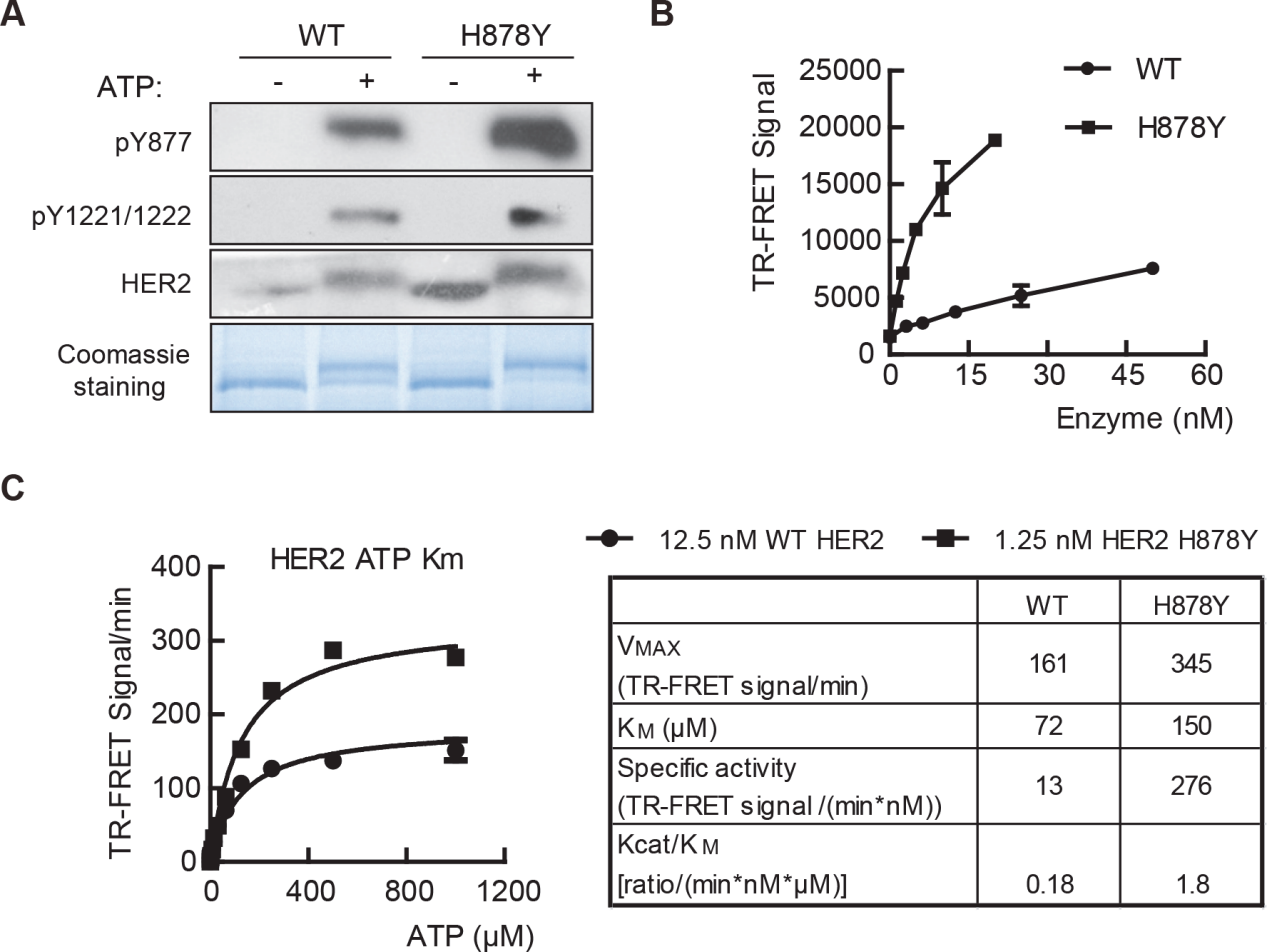
H878Y is a gain-of-function mutation. (A) H878Y mutant HER2 is more efficiently autophosphorylated. Purified HER2 fragments were autophosphorylated when incubated with ATP for 60 minutes and blotted with antibodies against HER2 pY877 and pY1221/pY1222 (upper panels). Proteins were incubated with ATP for 60 minutes and then separated with SDS-PAGE to visualize phosphorylated (upper band) and unphosphorylated proteins (lower band) (bottom panel). (B) Enzyme titration of wild type (WT) and H878Y isoforms. Enzymatic activity was assayed at 1mM ATP at room temperature for 1 hour. (C) Kinase activity assay using time-resolved fluorescence-resonance energy transfer (TR-FRET) methodology (left panel). WT and H878Y HER2 isoforms were assayed at various concentrations of ATP to determine the constants indicated in right panel.

We next sought to determine the molecular mechanism underlying the increased kinase activity of the H878Y isoform. Autophosphorylation of ErbB receptor family members is crucial for the activation of their tyrosine kinase activity. We therefore ask whether the H878Y isoform increases the autophosphorylation ability as compared to the WT isoform. To this end, the cytoplasmic domains of WT and H878Y HER2 were expressed in Sf21 cells using a baculovirus system and were subsequently purified for kinase assays. After onehour treatment with ATP using equal amounts of the purified kinases, the proteins were separated by SDS-PAGE (sodium dodecyl sulfate polyacrylamide gel electrophoresis) and the levels of autophosphorylation were assessed by western blot analysis. H878Y isoform exhibited increased levels of autophosphorylation on the Y1221/Y1222 residues. We also detected increased levels of phosphorylated Y877 (Fig 2A, upper panels), indicating that the H878Y mutant is autophosphorylated more efficiently than the wild type HER2. Consistently, Coomassie staining of SDS-polyacrylamide gels showed that after one hour incubation in ATP-containing reaction buffer, the H878Y isoform was phosphorylated to the degree that the corresponding unphosphorylated band was almost invisible (lower band due to faster moving speed in electrophoresis). In contrast, the unphosphorylated band was clearly visible for the WT isoform (bottom panel, Fig 2A). These data demonstrate that H878Y mutation increases the autophosphorylation of HER2.

As a receptor tyrosine kinase, kinase activity affects many aspects of HER2 function. We asked whether H878Y has enhanced kinase activity. Indeed, we found that the H878Y mutant exhibited significantly increased kinase activity compared to the WT enzyme (Fig 2B). To further characterize the enzymatic characteristics of the H878Y HER2 mutant, we measured the kinase activities of the both isoforms in the presence of various concentrations of ATP and calculated the Km of ATP and the maximal specific catalytic activity of these isoforms. Data showed that H878Y mutant exhibited a slightly increased ATP Km (150 μM vs. 72 μM) but a markedly increased specific catalytic activity (21-fold) compared to the WT enzyme (left panel, Fig 2C). More importantly, H878Y exhibit 10-fold higher the value of Kcat/Km compared to WT HER2 (right panel, Fig 2C).

Our data therefore demonstrated that H878Y mutation conferred higher kinase activity in comparison to WT HER2.

### Phosphorylated Y878 interacts with R898 to enhance the kinase activity of HER2

**Fig 3:**
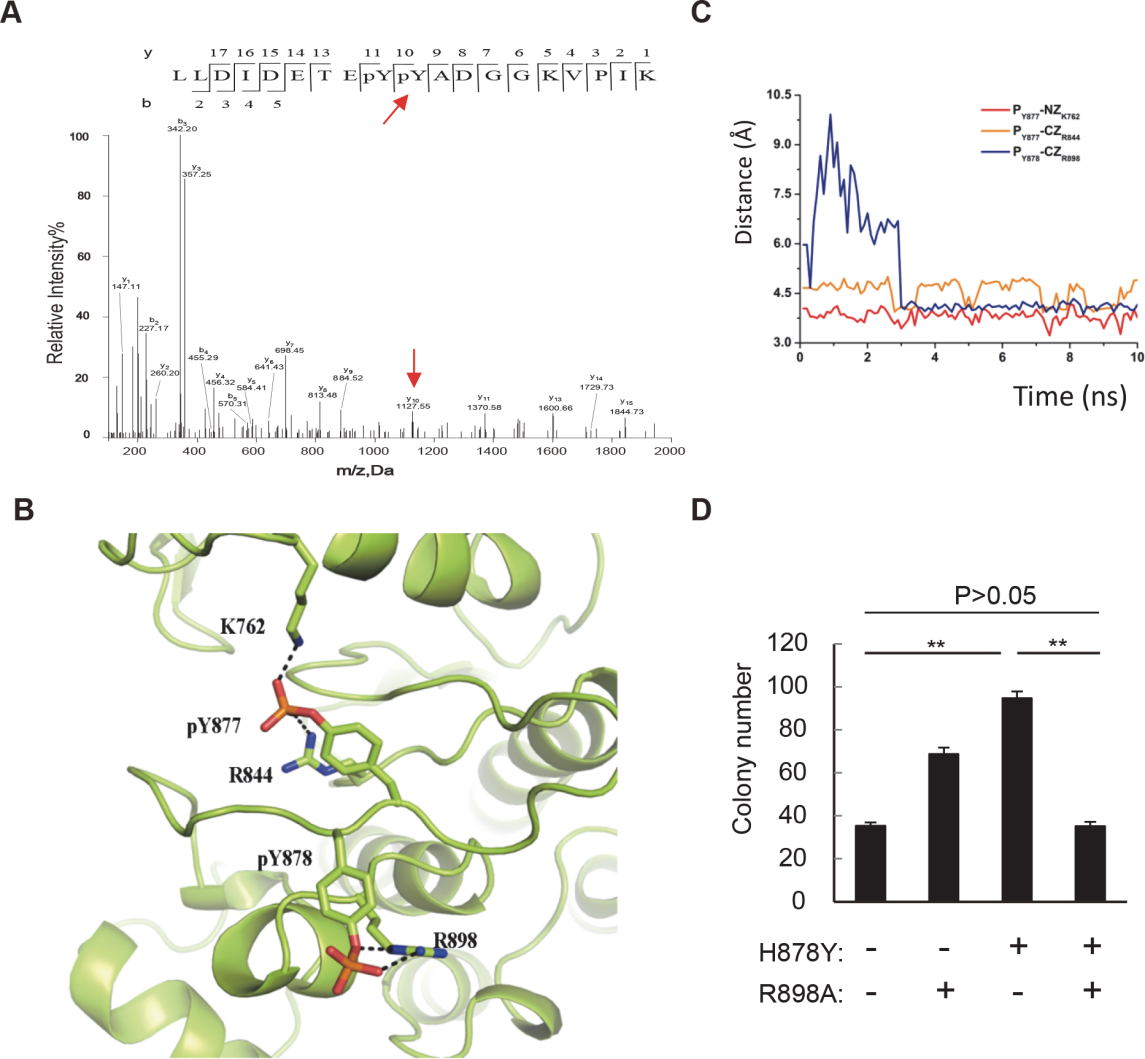
Y878 is phosphorylated to interact with R898 to enhance HER2 activity. (A) Mass spectrometry detection of Y878 phosphorylation. Red arrow indicates the phosphorylated Y878. (B) Molecular simulation on HER2 H878Y. Picture shows the last snapshot of HER2-pY878 mutant system, where pY878 forms salt bridge interactions with residue R898. (C) The distances of atom pairs pY877-NZK762 (colored in red), pY877-CZR844 (orange) and pY878-CZR898 (blue) in HER2-pY878 simulation. (D) Statistics of soft-agar assay on NIH-3T3 cells transfected with WT, R898A, H878Y, and H878Y/R898A HER2. H878Y/R898A double mutant HER2 transformed cells form similar colonies as WT HER2 on soft-agar assay. **P<0.01.

Phosphorylation of Y877 confers higher kinase activity of HER2 and correlates with activated states of HER2 [21]. Similar to Y877, the mutationally introduced Y878 locates in the activation loop. We next investigated whether Y878 can also be phosphorylated. Indeed, mass spectrometric data showed that Y878 is phosphorylated (Fig 3A and S1 Fig).

We further investigated how phosphorylated Y878 (pY878) increases the kinase activity of HER2. The crystal structure of the HER2 kinase domain has been previously resolved [22]. We thus modeled the structure of the phosphorylated H878Y mutant with both Y877 and Y878 in phosphorylated states. In our simulation, pY877 is spatially coordinated with the adjacent residues K762 and R844 (the average distances of the Y877-K762 and Y877-R844 pairs are 3.8 Å and 4.5 Å) and traps the kinase domain in an active conformation that is permissive for substrate binding (S1 Fig). Interestingly, Y878 might mimic the phosphorylation sites in the activation loop of other kinases, such as Y1007 and Y1008 in the JAK2 kinase [23]. In our 10-ns MD simulation, pY878 formed salt bridge interactions with the R898 residue (Fig 3B). The side chain of R898 was found to undergo a pronounced conformational rearrangement, as the distance of the Y878-R898 pair changed from 9.9 Å to 4 Å. In addition, the kinase domain of Y878 exhibited limited motion, with an average root-mean-square deviation (RMSD) of 2.1 Å during the last 1 ns of simulation, whereas the WT RMSD increased to 2.5 Å (Fig 3C and S1 Fig). Thus, we speculated that pY878 stabilized the active conformation of HER2.

To validate that pY878 enhances the kinase activity of HER2 by interacting with R898, we substituted R898 with alanine (R898A) to abrogate the formation of this potential salt bridge and assessed the resulting mutant kinase activity, as reflected by the colony formation ability of HER2-expressing cells in a soft-agar assay. The R898A HER2 mutant exhibited increased transforming ability, indicating that the R898A mutation by itself does not impair HER2 activity (Fig 3D). Interestingly, cells expressing the H878Y/R898A double mutant produced similar numbers of colonies as compared to cells expressing WT HER2 (*P* > 0.05, Fig 3D) but much less than cells expressing H878Y mutant, indicating that the R898A mutation counterbalances the increased activity conferred by the gain-of-function H878Y mutation. This result demonstrates that pY878 interacts with R898, resulting in increased HER2 kinase activity.

### H878Y mutant HER2 elicited signals are sensitive to HER2 inhibitor treatment

**Fig 4:**
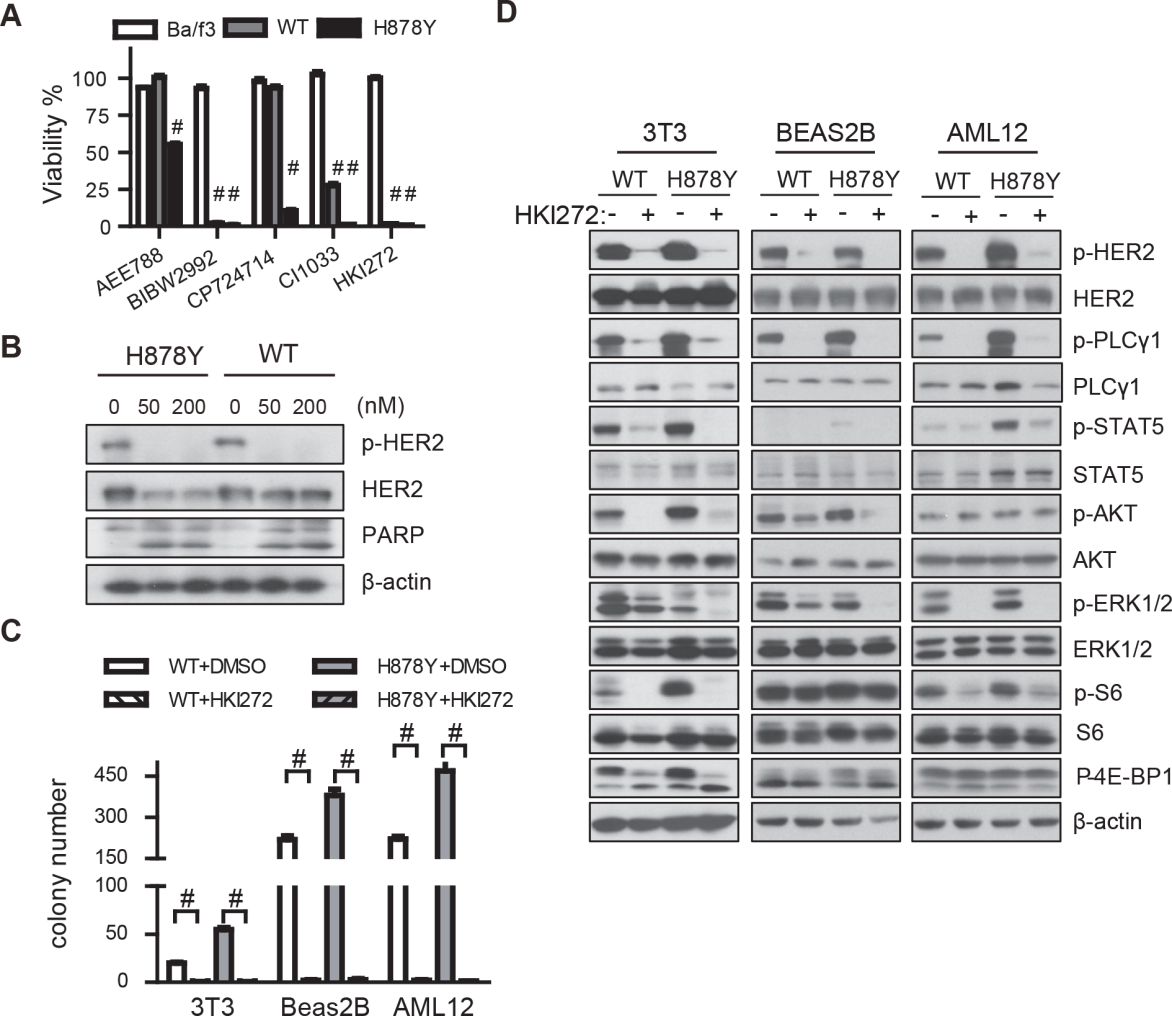
HKI-272 inhibits H878Y HER2 mutant elicited signaling. (A) WT and H878Y transformed Ba/f3 and parental cells were treated with 50nM of various HER2 inhibitors for 3 days to determine the viability. (B) WT and H878Y transformed Ba/f3 cells were treated with 50 or 200nM HKI-272 for 24 hours to determine HER2 phosphorylation and cleaved PARP. Western blot were carried out with antibody indicated. (C) HKI-272 efficiently eliminated soft-agar colonies of WT and H878Y transfected NIH-3T3, Beas-2B, and AML12 cells in the presence of 500nM HKI-272. (D) H878Y elicited signals are sensitive to HKI-272 inhibition. Control vector, WT and H878Y constructs transfected NIH-3T3, Beas-2B, and AML12 cells were treated with 500 nM HKI-272 for 1 hour and then subjected to Western blot analysis with antibodies indicated. # p<0.001.

Currently multiple HER2 inhibitors are being evaluated in various phases of clinical trial, and we ask whether these HER2 inhibitors (AEE788, BIBW2992, CP-724714, CI-1033 and HKI-272) are able to efficiently block H878Y mediated transformation capability. We first overexpressed the H878Y HER2 mutant in Ba/F3 cells and found that the transformants were no longer dependent on IL-3 for survival. Interestingly, the second-generation irreversible pan-ErbB inhibitors exhibited significant toxicity against the HER2-driven Ba/F3 cells (Fig 4A and S2 Fig). We next chose to work on HKI-272 as it demonstrated the highest efficacy and least toxicity to parental Ba/F3 cells, implicating that HKI-272 shows less side effect *in vivo*. HKI-272 efficiently eliminated phosphorylation of HER2 proteins and induced cleaved PARP (Fig 4B and S2 Fig) in these Ba/f3 cells. Importantly, HKI-272 efficiently eliminated colony forming ability of 3T3, Beas-2B, and AML12 cells transfected with either WT or H878Y HER2 (Fig 4C and S2 Fig), clearly indicating that HKI-272 was toxic to H878Y transformed cells.

We next studied how HKI-272 inhibited the signal transduction elicited by HER2. We found that HKI-272 efficiently eliminated phosphorylation of ectopically expressed WT and H878Y HER2 protein in all 3 cell background. Interestingly, although H878Y elicited stronger PLC-γ1, STAT5 and Akt than WT isoform, these downstream mediators were more sensitive to HKI-272 inhibition in H878Y and WT isoform expressing cells, suggesting that H878Y is a sensitizing mutation. We also observed inhibition of Erk phophorylation in response to HKI-272 administration and found that Erk phosphorylation in H878Y overexpressing-cells were more sensitive in all 3 cell background. Similarly, Akt was sensitive to HKI-272 inhibition in all 3T3 and Beas-2B cell background. To study signals further downstream, we checked S6 phosphoryation as surrogate for S6K activity and 4E-BP1, both being mTOR substrates that are controlled by Akt. Interestingly, we found that S6 phosphorylation was sensitive to HKI-272 inhibition in 3T3 and AML12 background, but not Beas-2B background. Instead, 4E-BP1 was sensitive to HKI-272 inhibition in 3T3, but not in AML12 background (Fig 4D). Our data therefore clearly showed that HKI-272 was able to inhibit H878Y mutant HER2 elicited signals and that HKI-272 was cytotoxic to H878Y transformed cell.

## Discussion

HCC therapy in clinic is limited by paucity of valid druggable targets. We here show that H878Y, a HER2 mutation recently identified in 11% of HCC patients, represents a gain-of-function mutation and that Y878 enhances the kinase activity of HER2 through a salt bridge interaction between pY878 and R898 that stabilizes the kinase in an active conformation. H878Y is transforming and transformed cell lines are sensitive to treatment with HKI-272.

Our work on the H878Y mutation has revealed a novel mechanism by which mutations can impact kinase activity. We demonstrate that the Y878 in HER2 mutant is phosphorylated and interacts with R898 to stabilize the active conformation of the kinase, thereby enhancing its activity. This mode of function mimics, to a certain extent, the Y1007 and Y1008 residues within the activation loop of the JAK2 kinase [23]. Tyrosine is frequently introduced into kinases via mutagenesis [24]. The EGFR H870Y, ITK D509Y and MST1 D175Y mutations were detected by sequencing, according to the COSMIC database (http://cancer.sanger.ac.uk/cancergenome/projects/cosmic/). However, the functional impact of these mutations remains to be characterized. The PDGFRa D842Y and D846Y mutations were reported to sensitize PDGFRa to imatinib [25]. Furthermore, the KIT D816Y [26], KIT D820Y [27] and FLT3 D835Y [28] mutations were reported to be activating mutations. However, the molecular mechanism underlying their effects remains unknown. The mechanism underlying the increased kinase activity of the H878Y HER2 mutant might therefore be generally implicated in the altered function of the many mutant kinases.

H878Y mutation is a gain-of-function mutation that is capable of transforming NIH-3T3, Ba/F3 Beas-2B, and AML12 cells. Interestingly, we found that H878Y is a sensitizing mutation for HER2 kinase inhibitors, consistent with a previous report [29]. Therefore, the targeting of this mutant represents a valid therapeutic modality for cancer patients of this genotype. This is of particular interest because few driver oncogenes have been verified for liver cancer patients in the clinic.

In summary, our work clearly shows that H878Y, a gain-of-function mutation found in a portion of HCC patients, positively impact on kinase activity of HER2 through phosphorylation of this mutationally introduced tyrosine to impact on structure of HER2 kinase domain.

## Supporting Information

S1 Fig Biochemical characterization of H878Y mutant HER2. (A) Mass spectrometry detection of Y878 phosphorylation. A single phosphorylation site on Y878, on the background of unphosphorylated Y877 is detected by mass spectrometry. (B) The last snapshot of HER2-WT in 10 ns MD simulation, where pY877 coordinating with residues K762 and R844. (C) Comparison of the RMSDs in 10 ns simulations of HER2-WT (black), HER2-pY878 (red). The RMSDs were calculated over the Cα atoms of kinase domain with respect to the crystal structure.
(TIF)

S2 Fig H878Y is sensitive to HER2 inhibitors. (A), (B), WT and H878Y transformed Ba/f3 and parental cells were treated with 200nM of various HER2 inhibitors for 3 days to determine the viability, n = 6 (A); or for 4 hours to probe HER2 downstream signaling (B). D,DMSO; H,HKI-272; B, BIBW2992; C, CI1033. (C) Viability of Ba/F3 cells transformed by WT or H878Y mutant HER2. 2×10^3^ cells were treated with HER2 inhibitors for 3 days, cell viability were determined by CellTiter-Glo luminescent cell viability assay. n = 8. (D) WT and H878Y transformed Ba/f3 cells were treated with 50nM of various HER2 inhibitors for 12 hours, immunoblots of HER2 signaling were shown. D,DMSO; H,HKI-272; B, BIBW2992; C, CI1033; CP, CP724714. (E) Colony formation assay. Vector, WT or H878Y transfected AML12 cells (1×10^5^ cells) were treated with 500nmM of HKI-272 for 4 days, cells were fixed and stained with 0.5% crystal violet.
(TIF)

S1 Protocol Supplementary materials and methods. (DOC)
