# Supplementary figures and images for: Phosphorylation of Mutationally Introduced Tyrosine in the Activation Loop of HER2 Confers Gain-of-Function Activity
Source: PLoS One. 2015 Apr 8;10(4):e0123623. doi: 10.1371/journal.pone.0123623 (PMC4390223; doi:10.1371/journal.pone.0123623)

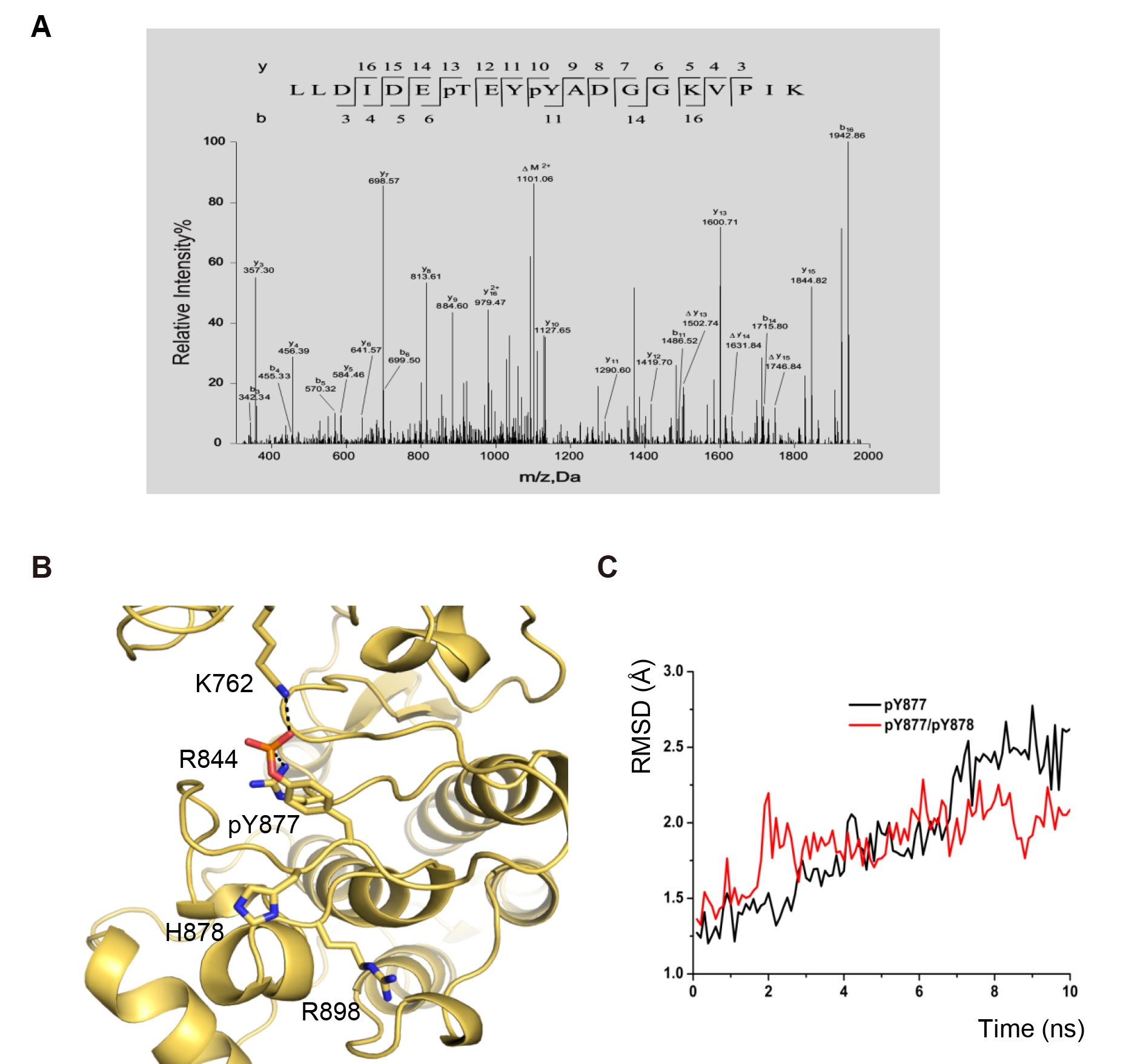

Supplement: S1 Fig — (A) Mass spectrometry detection of Y878 phosphorylation. A single phosphorylation site on Y878, on the background of unphosphorylated Y877 is detected by mass spectrometry. (B) The last snapshot of HER2-WT in 10 ns MD simulation, where pY877 coordinating with residues K762 and R844. (C) Comparison of the RMSDs in 10 ns simulations of HER2-WT (black), HER2-pY878 (red). The RMSDs were calculated over the Cα atoms of kinase domain with respect to the crystal structure. (TIF) [file pone.0123623.s001.tif]

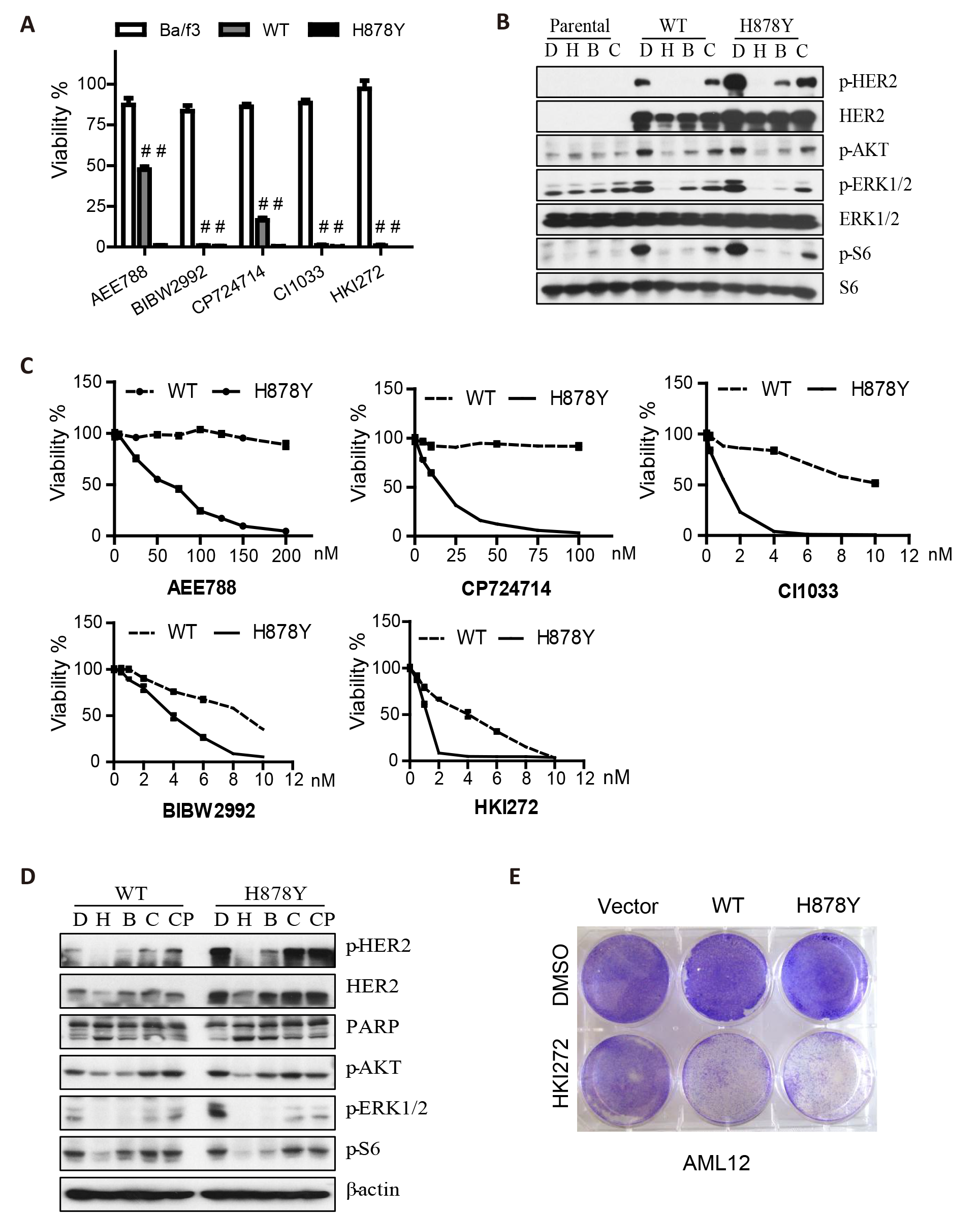

Supplement: S2 Fig — (A), (B), WT and H878Y transformed Ba/f3 and parental cells were treated with 200nM of various HER2 inhibitors for 3 days to determine the viability, n = 6 (A); or for 4 hours to probe HER2 downstream signaling (B). D,DMSO; H,HKI-272; B, BIBW2992; C, CI1033. (C) Viability of Ba/F3 cells transformed by WT or H878Y mutant HER2. 2×10^3^ cells were treated with HER2 inhibitors for 3 days, cell viability were determined by CellTiter-Glo luminescent cell viability assay. n = 8. (D) WT and H878Y transformed Ba/f3 cells were treated with 50nM of various HER2 inhibitors for 12 hours, immunoblots of HER2 signaling were shown. D,DMSO; H,HKI-272; B, BIBW2992; C, CI1033; CP, CP724714. (E) Colony formation assay. Vector, WT or H878Y transfected AML12 cells (1×10^5^ cells) were treated with 500nmM of HKI-272 for 4 days, cells were fixed and stained with 0.5% crystal violet. (TIF) [file pone.0123623.s002.tif]
