# Supplementary material for: Phosphorylation of Mutationally Introduced Tyrosine in the Activation Loop of HER2 Confers Gain-of-Function Activity
Source: PLoS One. 2015 Apr 8;10(4):e0123623. doi: 10.1371/journal.pone.0123623 (PMC4390223; doi:10.1371/journal.pone.0123623)
Supplement: S1 Protocol — (DOC) [file pone.0123623.s003.doc]

**Supplementary materials and methods**

**Protein purification and enzyme activity assay**

For enzyme assays, cytoplasmic domain (amino acids 676-1255) of human HER2 (wild-type, H878Y) were subcloned into the pFastBac1 vector (Invitrogen) with a N-terminal 6 x His tag. The constructs were transfected into Sf21 cells according to manufacturer’s standard protocol. The expressed protein were purified using Chelating Sepharose (GE).The activities of HER2 kinase domains were tested in an assay based on the time-resolved fluorescence-resonance energy transfer (TR-FRET) methodology in a reaction mixture containing HER2 kinases, 1 mMATP, biotinylated peptide substrate (CisBioBioassys) reconsituted with 50 mM HEPES pH7.5, 10 mM MgCl_2_, 1 mM EGTA, 0.01% Brij-35, 2.5 mM DTT and 0.1% BSA. Concentrations of HER2 kinase were indicated in Figure 1 and the reaction was initiated by the addition of ATP and substrate. After reaction at room temperature for 60 minutes, an equal volume of stop/detection solution containing Eu^+^cryptate conjugated antiphosphotyrosine antibody (CisBioBioassys) and Streptavidin-XL665 (CisBioBioassys) in buffer with 25 mM Tris pH7.4, 400 mM KF, 50 mM EDTA, 0.01% BSA, 0.01% Triton X-100 was added according to the manufacture’s instruction (CisBio Bioassays). Plates were sealed and incubated at room temperature for 1 hour, and the TR-FRET signals (ratio of fluorescence emission at 665 nm over emission at 620 nm with excitation at 337 nm wavelength) were recorded on a PHERAstar FS plate reader (BMG Labtech). Phosphorylation of biotinylated peptide substrate led to the binding of both Eu^+^cryptate conjugated antiphosphotyrosine antibody and streptaviding-XL665 to the biotinylated peptide. The close proximity of the donor (Eu^+^cryptate ) and acceptor (XL665) fluorophore led to a high degree of fluorescenceresonance energy transfer from the donor fluorophore (at 620 nm) to the acceptor fluorophore (at 665 nm). Kinetics parameters for three HER2 kinases were determined in the same TR-FRET assays with fixed enzyme concentrations and various ATP concentrations. The reaction time was 30 minutes. K_m_ was derived from fitting the ATP titration data to the Michaelis-Menten equation by Graphpad Prism software.

Michaelis-Menten equation: Y=V_max_*X/(K_m_+X) (X represents the ATP concentration; Y the reaction velocity at X; V_max_ is the top of the curve effect; K_m_ is the ATP concentration corresponding to half signal value of V_max_). Specific activity is derived from the equation: Specific activity =Vmax/[Enzyme] ([Enzyme] represents the enzyme concentration).

**HER2 autophosphorylation**

Purified protein (wild-type HER2 and HER2 H878Y) were incubated with 1mM ATP and biotinylated peptide substrate at room temperature for 1 hour. Then reactions were stopped by the addition of 5×SDS-PAGE loading buffer. Non-incubated samples were used as controls. All samples were applied for western blotting and Mass spectrometric analysis.

**Mass spectrometric analysis**

Protein bands on the SDS-PAGE gel were de-stained, and then reduced in 10 mM DTT at 56 °C for 30 min followed by alkylation in 55 mM iodoacetamide at dark for 1 hr. After that the protein bands were in-gel digested with sequencing grade trypsin (10 ng/μL trypsin, 50 mM ammonium bicarbonate, pH 8.0) overnight at 37℃. Peptides were extracted with 5% formic acid/50% acetonitrile and 0.1% formic acid/75% acetonitrile sequentially and then concentrated to ~ 20 μl. The extracted peptides were separated by an analytical capillary column (50 μm× 10 cm) packed with 5 μm spherical C18 reversed phase material (YMC). A Waters nanoAcquity UPLC system (Waters) was used to generate the following HPLC gradient: 0-30% B in 40 min, 30-70% B in 15 min (A = 0.1% formic acid in water, B = 0.1% formic acid in acetonitrile). The eluted peptides were sprayed into a LTQ OrbitrapVelos mass spectrometer (ThermoFisher Scientific) equipped with a nano-ESI ion source. The mass spectrometer was operated in data-dependent mode with one MS scan followed by four CID (Collision Induced Dissociation) and four HCD (High-energy Collisional Dissociation) MS/MS scans for each cycle. Database searches were performed on an in-house Mascot server (Matrix Science Ltd.) against HER2 receptor protein sequence. The search parameters are: 7 ppm mass tolerance for precursor ions; 0.5 Da mass tolerance for product ions; three missed cleavage sites were allowed for trypsin digestion and the following variable modifications were included: oxidation on methionine, carbamidomethylation on cysteine, phosphorylation on serine, threonine, and tyrosine. The tandem mass spectra of matched phosphorylated peptides were manually checked for their validity.

**MD simulation**

The mutants of phosphoryalted H878Y and WT (phosphoryalted Y877) were directly generated based on HER2 crystal structure . Their side chains were predicted using Protein Local Optimization Program (PLOP) in variable dielectric model . The molecular dynamics simulation was performed using program Desmond (version 2.2.7.3.0) with OPLS 2005 force field in 0.15 M NaCl and TIP4P explicit water model. The cubic boundary condition was selected and no protein atom was within 12 Å of the edge. The whole system contains about 46,300 atoms and is 78 × 78 × 78 Å^3^ in size. The equilibration of solvated system was performed with 2,000 steps of steep descent minimization followed by 3,000 steps of L-BGFS minimization, with 50 kcal·mol^-1^ ·Å ^-2^ harmonic position restraints applied to heavy atoms of the solute. The production run was performed in MTK NPT (1 bar, 300 K) ensemble for 10 ns. The cutoffs of short-range electrostatic and Lennard-Jones interactions were 10 Å. Long-range electrostatic interactions were computed by the Particle Mesh Ewald method using 64×64×64 grid with σ = 2.18 Å . The M-SHAKE algorithm was used to constrain all bonds involving hydrogen atoms with the integration step size of 2 fs.

The root-mean-squared deviations (RMSDs) of HER2 Cα atoms were calculated after superimposing to the crystal structure. The distance of residue pairs Y877-K762 (atoms P-N_Z_), Y877-R844 (atoms P-C_Z_), Y878-R896 (atoms P-C_Z_), Y878-R898 (atoms P-C_Z_) were measured respectively. The structural figure was drawn in PyMOL ([www.pymol.org](http://www.pymol.org/)).

**References**

1. Aertgeerts K, Skene R, Yano J, Sang BC, Zou H, Snell G, et al. Structural analysis of the mechanism of inhibition and allosteric activation of the kinase domain of HER2 protein. Journal of Biological Chemistry. 2011;286(21):18756-65. doi: 10.1074/jbc.M110.206193

PubMed PMID: 21454582; PubMed Central PMCID: PMC3099692.

2. Jacobson MP, Kaminski GA, Friesner RA, Rapp CS. Force field validation using protein side chain prediction. Journal of Physical Chemistry. 2002;106(44):11673-80.

3. Li X, Jacobson MP, Friesner RA. High-resolution prediction of protein helix positions and orientations. Proteins. 2004;55(2):368-82. doi: 10.1002/prot.20014. PubMed PMID: 15048828.

4. Huang N, Kalyanaraman C, Irwin JJ, Jacobson MP. Physics-based scoring of protein-ligand complexes: enrichment of known inhibitors in large-scale virtual screening. Journal of Chemical Information and Modeling. 2006;46(1):243-53. doi: 10.1021/ci0502855. PubMed PMID: 16426060.

5. Bowers KJ, Chow E, Xu H, Dror RO, Eastwood MP, Gregersen BA, et al., editors. Scalable algorithms for molecular dynamics simulations on commodity clusters. Proceedings of the 2006 ACM/IEEE conference on Supercomputing 2006; Tampa, Florida. 1188544: ACM.

6. Jorgensen WL, Maxwell DS, Tirado-Rives J. Development and testing of the OPLS all-atom force field on conformational energetics and properties of organic liquids. Journal of the American Chemical Society. 1996;118(45):11225-36. doi: 10.1021/ja9621760.

7. Jensen KP, Jorgensen WL. Halide, ammonium, and alkali metal ion parameters for modeling aqueous solutions. Journal of Chemical Theory and Computation. 2006;2(6):1499-509. doi: 10.1021/ct600252r.

8. Jorgensen WL, Madura JD. Temperature and size dependence for Monte Carlo simulations of TIP4P water. Molecular Physics. 1985;56(6):1381-92.

9. Darden T, York D, Pedersen L. Particle mesh Ewald: An N • log (N) method for Ewald sums in large systems. Journal of Chemical Physics. 1993;98:10089.

10. Kräutler V, van Gunsteren WF, Hünenberger PH. A fast SHAKE algorithm to solve distance constraint equations for small molecules in molecular dynamics simulations. J Comput Chem. 2001;22(5):501-8. doi: 10.1002/1096-987x(20010415)22:5<501::aid-jcc1021>3.0.co;2-v.
